# Temporal Sequences of Synapse Disintegration Triggered by Afferent Axon Transection, Time-Lapse Imaging Study of Presynaptic and Postsynaptic Molecules

**DOI:** 10.1523/ENEURO.0459-18.2019

**Published:** 2019-10-02

**Authors:** Takusei Cho, Yutaro Kashiwagi, Shigeo Okabe

**Affiliations:** Department of Cellular Neurobiology, Graduate School of Medicine, the University of Tokyo, Tokyo 113-0033, Japan

**Keywords:** dendritic spine, hippocampus, live imaging, NMDA receptor, synaptic plasticity, traumatic brain injury

## Abstract

Traumatic brain injury (TBI) is one of the major causes of death and disability. Multiple animal models have been developed to explore therapeutic targets for TBI. However, heterogeneity of pathophysiology obstructs discovery of therapeutic targets. To facilitate understanding of TBI pathophysiology, each element of neuronal and glial responses should be studied separately. We focused on synapse remodeling which plays an important role in recovery from TBI and developed a new method, afferent elimination, for analyzing synapse remodeling after selective damage to presynaptic axons by mechanical transection in culture of mouse hippocampal neurons. Afferent elimination can induce various events related to synapse remodeling and we could determine their temporal orders and find relationships between them. Specifically, loss of presynaptic sites preceded loss of postsynaptic sites and spines. Some of the postsynaptic sites initially located inside spines showed translocation toward dendritic shafts. These translocation events started after the loss of contacting presynaptic sites. Also, these events could be blocked or delayed by NMDA receptor inhibition. Taken together, these findings suggest that postsynaptic changes occur in afferent elimination are NMDA dependent and imply that these NMDA-dependent events underlie synaptic remodeling of TBI.

## Significance Statement

Traumatic brain injury (TBI) is one of the major causes of death and disability. However, heterogeneity of TBI pathophysiology obstructs discovery of therapeutic targets. To solve this, each element of neuronal and glial responses should be studied separately. We focused on synapse remodeling which plays an important role in recovery from TBI and developed a new method, afferent elimination. Afferent elimination can induce various events related to synapse remodeling and we could determine their temporal orders and find relationships between them. Also, these events could be blocked by NMDA receptor inhibition. Taken together, these findings suggest that postsynaptic changes after afferent elimination are NMDA receptor-dependent and imply that NMDA receptor-related signaling underlies synaptic remodeling in TBI.

## Introduction

Traumatic brain injury (TBI) is a major cause of death and disability in multiple countries and contributes to ∼30% of all injury deaths in the United States (Taylor et al., 2017). It is important to propose new therapeutic strategies for the improvement of survival rate and reduction of disability, not only prevent TBI itself. Multiple animal models that replicate human TBI have been developed for better understanding of TBI pathophysiology and exploring therapeutic targets (Xiong et al., 2013). However, promising drugs which were effective in TBI models have all failed in clinical trials for human (Xiong et al., 2013). One of the causes of the failure is heterogeneity of pathophysiology. Many pathologic events occur after TBI, such as changes in cellular conditions like ionic balance, glucose metabolism and free radical generation (Prins et al., 2013), together with morphologic changes including axonal degeneration and synapse elimination (Park and Biederer, 2013). Neuronal circuits damaged by TBI also start multiple programs for functional recovery and regeneration, such as synaptic plasticity, neurogenesis, gliogenesis, and axonal sprouting (Schoch et al., 2012; Nudo, 2013). To solve the complex mechanisms during the recovery process, each element of neuronal and glial responses should be studied separately.

In the process of recovery from TBI, remodeling of synapses may play important roles for both the generation of new compensatory neural network and reconnection of the lost network. In many pathologic conditions, long axonal tracts are damaged and degeneration of axons projecting into specific brain regions takes place, while leaving postsynaptic neurons intact (Brody et al., 2015). In postsynaptic intact neurons, compensatory formation of new synapses should occur in parallel with elimination of synapses triggered by TBI (Nahmani and Turrigiano, 2014; Bandaru et al., 2015). Therefore, time course and extent of synapse elimination should affect *de novo* formation of new synapses, which are important in functional recovery of the neural circuits from the damage. However, there have been little information about the relationship between synapse elimination and synapse regeneration. Another important question in post-traumatic synapse remodeling is whether postsynaptic components in the intact dendrites are taken over by nearby intact axons. Previous *in vivo* two-photon imaging of spine formation in the adult brain revealed maintenance of newly formed spines without presynaptic contacts for a couple of days (Knott et al., 2006). If spines can be maintained without presynaptic partners for a long time after traumatic injury, the chance of postsynaptic spines to find new synaptic partners should increase. To answer these questions related to synapse remodeling after TBI, an appropriate *in vitro* model is necessary. However, there have been few reduced culture systems that allow selective manipulation of presynaptic axons without affecting postsynaptic neurons (Morrison et al., 2011).

In this study, we developed a new method of analyzing synapse remodeling after selective damage to presynaptic axons. The protocol of manually severing incoming axons (afferent elimination) to a single postsynaptic target neuron effectively removed most of the presynaptic structures associated with intact postsynaptic dendrites. Dynamic changes in both presynaptic and postsynaptic molecules could be analyzed by fluorescent probes for presynaptic and postsynaptic molecules. Afferent elimination also induced reduction in the number of postsynaptic densities (PSDs), which were detected by fluorescently tagged PSD-95. Time-lapse imaging revealed temporal orders of synapse elimination, with disappearance of presynaptic components first, followed by deconstruction of postsynaptic components. Dual imaging of presynaptic and postsynaptic molecules frequently detected movements of PSDs toward dendritic shafts, simultaneously with spine shrinkage. These morphologic changes in the postsynaptic components were triggered by disappearance in presynaptic components detected by fluorescently tagged synaptophysin. Moreover, administration of DL-amino-5-phosphonovalerate (AP5), a NMDA receptor antagonist, could not stopped but delayed disappearance of PSD-95-positive spines. The *in vitro* assay based on afferent elimination is a simple but reliable system for the assessment of temporal pattern in postsynaptic responses to presynaptic axon damage.

## Materials and Methods

### Plasmid and adenovirus vectors

Plasmid vectors for the expression of GFP or PSD-95-TagRFP under the control of β-actin promoter were used in this study. Recombinant adenovirus expressing PSD-95-GFP, PSD-95-CFP, and YFP-Homer1c under the control of CAG promoter was reported previously (Okabe et al., 1999, 2001; Kuriu et al., 2006). Synaptophysin-YFP was kindly provided from Nobutaka Hirokawa (The University of Tokyo).

### Hippocampal primary culture

All animal experiments were approved by the animal welfare ethics committee of the University of Tokyo. Dissociated primary hippocampal cultures were prepared from E16.5 ICR mouse embryos of either sex as described previously with minor modifications (Okabe et al., 1999). First, hippocampi were treated with trypsin (Gibco) and DNase (Sigma). Then, they were mechanically dissociated and suspended in MEM containing B18 supplement, L-glutamine (Gibco), and 5% FCS (Equitech-Bio). After preparation of cell suspensions, they were plated onto a glass-bottom dishes (MatTek) coated with poly-L-lysine (Sigma). To prevent glial cell proliferation, 5 μM ara-C (Sigma) was added to cultures 2 d after plating.

### Gene transfection and adenovirus infection

Ca^2+^-phosphate transfection was performed after 8–9 d *in vitro* according to a standard procedure. Briefly, plasmid vectors were mixed with 2× phosphate buffer and incubated at room temperature for 15 min to generate a calcium phosphate-DNA co-precipitate. The medium was replaced with transfection medium and the calcium phosphate-DNA co-precipitate was added to dishes. Cells were incubated for 50 min in a 5% CO_2_ incubator at 37°C and were returned to the original medium until the following experiments.

Hippocampal cultures were expose to the virus at 11–12 d *in vitro*. Medium was not changed during virus exposure. Cells were incubated at least 2 d to allow the cells to express enough amounts of fluorescent proteins.

### Afferent axon cutting

Axon cutting was performed at 13–15 d *in vitro*. Live cells with or without expressing fluorescent probes were placed on the stage of an inverted microscope. A target postsynaptic neuron was selected under the microscope. The target cell was selected based on the distance between the somata of the target neuron and the nearest neighbor neuron, which should be >100 µm, and little overlap of their dendrites. For time-lapse fluorescence imaging, we selected cells that express postsynaptic markers. The culture surface was scratched with sharp-pointed forceps to generate a circular zone, which is free of incoming axonal structures, is >50 µm away from the distal ends of dendrites grown from the target postsynaptic neuron, and completely encircle the target neuron. Trajectory of surface scratching by forceps was designed to minimize direct mechanical interaction with cell bodies and thick dendrites of surrounding cells. We tried to minimize scratching of cell bodies and dendrites, as this tended to dislocate these structures and induce deformation of axons inside the cutting line. The diameter of the intact circular area inside the cutting line was 250–400 µm. All procedures were finished within 15 min per dish, and the cells were viable at least for 2 d. If cells after this manipulation were used for live-imaging, they were placed at least for 3 h in incubators to allow recovery from damage. All the steps of afferent axon cutting were carefully monitored under the phase-contrast microscope and any preparations that showed the sign of cell injuries outside of the manipulated area were discarded.

For more precise control of the trajectory of surface scratching, we used an epi-fluorescence microscope with phase-contrast illumination and a micromanipulator controlling a needle with its tip diameter of 0.30 mm. This system was effective in manipulations that preserved the axons growing from the target postsynaptic neuron and removed the other afferent axons as much as possible. The postsynaptic target neurons were labeled by GFP and PSD-95-TagRFP. GFP fluorescence of the axons was monitored during the cutting procedure to keep the axonal structure intact. Cells were observed before and 24 h after cutting procedure.

### Pharmacology

Neurons were treated with 1 µM TTX (Wako), 50 µM DL- AP5 (Sigma), or 10 µM CNQX (Tocris). All drugs were administrated directly into dishes containing the medium just after axon cutting procedure. After drug administration, cells were returned to incubators until the following experiments.

### Immunocytochemistry

Hippocampal neurons were fixed in 2% paraformaldehyde in PBS for 25 min. After fixation, cells were treated with 0.2% Triton X-100 in PBS for 5 min, blocked with 5% NGS, and incubated with mouse monoclonal anti-PSD-95 (1:100; Invitrogen), rabbit monoclonal anti-PSD-95 (1:200; Cell Signaling), mouse monoclonal anti-bassoon (1:500; Enzo Lifescience), rabbit polyclonal anti-neurofilament 200 (1:250; Sigma) and chicken polyclonal anti-MAP2 (1:500; Phosphosolutions). Primary antibodies were visualized with goat anti-mouse, anti-rabbit or anti-chicken IgG conjugated to Alexa Fluor 488, Alexa Fluor 546 or Alexa Fluor 633 (1:500; Invitrogen).

### DiI staining

Hippocampal neurons were fixed in 2% paraformaldehyde in PBS for 25 min. Individual cells were labeled with 1,1'-dioctadecyl-3,3,3',3'-tetramethylindocarbocyanine perchlorate (DiI; Invitrogen) dissolved in a sunflower seed oil (Wako) and applied onto cell bodies by pressure ejection with a FemtoJet (Eppendorf). The cells were left on the stage at least for 30 min at room temperature to allow the dye to spread, then DiI was washed out by exchange of PBS.

### Live cell staining

Stock solution of propidium iodide (PI; Dojindo) was added directly to the culture medium to its final concentration of 6 µM. Cells were incubated with PI for 15 min in a 5% CO_2_ incubator at 37°C and fixed in 2% paraformaldehyde in PBS for 25 min. Cells were observed immediately after fixation.

### Microscopy

Images were obtained using a confocal microscope system (FV1000, Olympus) equipped with a 60× oil immersion lens (NA 1.42, Olympus) and 488, 561, 640 nm lasers. Images were collected at an additional electronic zoom factor of 7× (DiI and immunostaining) or 5× (GFP and PSD-95-TagRFP) and multiple optical slices with 0.5–1 µm in *z*-steps and the total thickness of 5–10 µm.

Live cells were placed in a custom-made chamber at 37°C with a continuous flow of 5% CO_2_ to maintain pH of the medium. To prevent evaporation of the medium, dishes were covered by a custom-made lid which can pass air and CO_2._ Live cell imaging was performed with a custom-made fluorescent microscope system based on an inverted microscope (IX81, Olympus), equipped with a 100× oil immersion lens (NA 1.49, Olympus), a Z-Drift Compensation System (Olympus), a motorized XY stage and an EMCCD camera (iXon3, Andor). MetaMorph software (Universal Imaging) was used to control filter wheels and *z*-axis controller. Light from a metal halide lamp (PhotoFluor II, Chroma) was passed through single band exciters, reflected by a dichroic mirror (Di01-R405/488/561/635, Semrock). Fluorescence signal was detected by the camera operated with EM Gain. Single XY images were obtained with intervals of 5 min from 3 to 15 h after axon cutting.

### Data analysis

Digital images were analyzed using ImageJ (NIH). Maximal intensity projection images were generated for each image stack and used for analysis. Spines were defined as protrusions which are <3 µm in length measured from dendritic shafts and the number of spines was counted manually.

For identification of the fluorescent clusters, the level of background fluorescence was first determined and their numbers were counted manually. PSD-95-GFP clusters in experiments with adenovirus infection were judged as dendritic PSD-95 if the cluster positions were at the edge of the dendrites. PSD-95-TagRFP clusters in experiments of co-expression with GFP or immunostained PSD-95 clusters in experiments of co-staining with DiI were judged as dendritic PSD-95 if the clusters were inside of spines the counter of which determined from the images of GFP or DiI. Immunopositive clusters of PSD-95 and bassoon in experiments of co-immunostaining with MAP2 were counted by the following methods: first, images were smoothed by ImageJ and processed by low-pass filters. Then, thresholds were set manually and clusters consisted of 5 or more pixels were counted by “Analyze Particles” plugin for ImageJ.

For analysis of PSD-95-CFP clusters with synaptophysin-YFP clusters, two clusters were judged to be at the same synaptic sites if one or more pixels of two clusters overlapped.

For correlative fluorescence imaging of DiI and immunostaining, cells were first stained with DiI and imaged by fluorescence microscopy. Then, immunostaining was performed with a standard procedure as mentioned above. Maximum intensity projection of the fluorescent images was made separately, and the images were aligned by MultiStackReg plugin for ImageJ.

### Statistics

For statistical analysis, an unpaired or paired Student’s *t* test was performed with Excel (Microsoft), ANOVA (two-way) and *post hoc* multiple comparison tests (Bonferroni and Tukey) were performed with GraphPad Prism 6 (GraphPad Software). The results of statistical tests used for each experiment were given with *p* values in Results. Type-1 error rates for all tests were set at 0.05. Error bars in figures represent the SEM.

## Results

### Loss of presynaptic elements induced by afferent elimination

**Figure 1.**
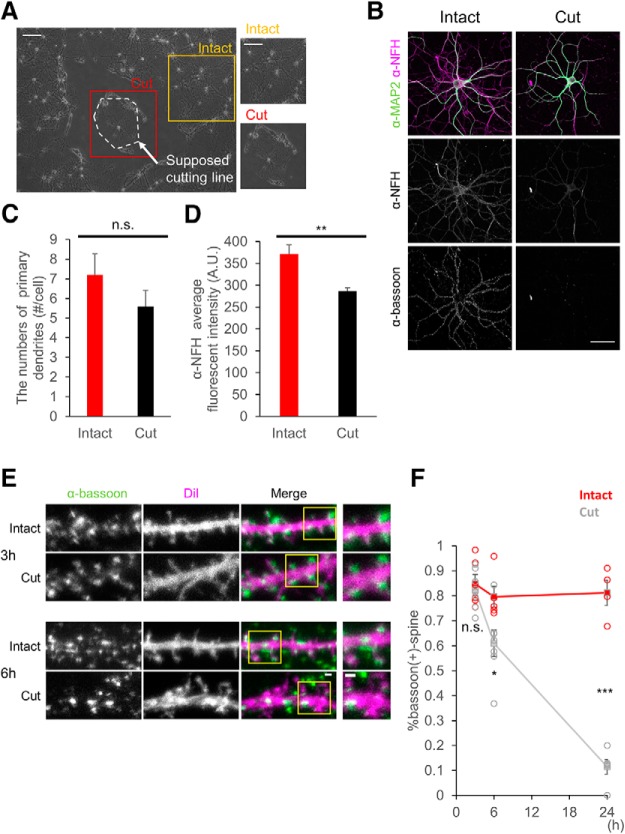
Time course of loss of presynaptic sites after afferent axon cutting. ***A***, Phase-contrast images of intact cells and a target cell with afferent elimination. Two images on the right side show regions inside orange (intact) and red (cut) squares. ***B***, Confocal images of dendrites grown from an intact cell or a cell with afferent elimination 24 h after axon manipulation. Top images show a dendritic marker anti-MAP2 staining (green) and a dendrite-axon marker anti-neurofilament 200 (NF-H) staining (magenta). Middle images show anti-NF-H. Bottom images show anti-bassoon staining, a marker of presynaptic active zones. ***C***, The numbers of primary dendrites 24 h after afferent elimination. ***D***, Average fluorescence intensity of NF-H in individual images. ***E***, Confocal images of dendrites in intact cells and cells 3 and 6 h after afferent elimination. Columns shows the anti-bassoon staining, DiI fluorescence, merged images of anti-bassoon (green) and DiI (magenta), and magnified images of regions inside yellow squares from left to right, respectively. ***F***, Fractions of bassoon-positive spines in total spine population 3, 6, and 24 h after afferent elimination. Error bars are SEM; *p < 0.05, **p < 0.01, ***p < 0.001, n.s. = not significant. Scale bars = 200 µm (***A***), 50 µm (***B***), 1 μm (***E***). Additional data about the damage to the isolated cells in afferent elimination can be found in Extended Data Figure 1-1.

We established an experimental procedure, afferent elimination, which removes almost all presynaptic boutons that connect with a single postsynaptic target neuron in culture, by inducing degeneration of incoming axon fibers by mechanical transection (Fig. 1*A*). Anti-neurofilament 200 (NF-H) antibody staining of the culture preparations 24 h after afferent elimination confirmed reduction of NF-H immunoreactivity (∼80% of the control areas) specifically within the axon transection zones (Fig. 1*B*,*D*; intact: 371.8 ± 20.4, *n* = 5 cells; cut: 286.4 ± 8.0/cell, *n* = 5 cells; *p* = 0.004, unpaired *t* test was used). To evaluate the dendritic morphology of the target postsynaptic neurons after afferent axon cutting, anti-MAP2 immunostaining was performed. We confirmed intact MAP2-positive dendritic morphology and the comparable average number of primary dendrites after afferent elimination (Fig. 1*B*,*C*; intact: 7.2 ± 1.1/cell, *n* = 5 cells; cut: 5.6 ± 0.8/cell, *n* = 5 cells; *p* = 0.267, unpaired *t* test). Transection of axons from the postsynaptic target neuron may induce traumatic damage, including acute increase in membrane permeability (LaPlaca et al., 2009). To test this possibility, we monitored GFP fluorescence in the postsynaptic target neurons at 3, 6, and 24 h after afferent elimination and found any detectable decrease of GFP fluorescence, indicating the intact plasma membrane after manipulation (Extended Data Fig. 1-1*A*,*B*). The distal part of axons from the target neurons was degenerated after transection (Extended Data Fig. 1-1*A*,*B*). On the other hand, the proximal part of the axons was intact in multiple trials of the axon transection and subsequent NF-H immunostaining for labeling GFP-positive axonal structure extending from the isolated neurons (*n* = 3 trials; Extended Data Fig. 1-1*C*,*D*). These results were consistent with the idea that our procedure of afferent elimination significantly reduced local axonal components without major direct mechanical insults to the postsynaptic neurons.

10.1523/ENEURO.0459-18.2019.f1-1 Extended Data Figure 1-1. Evaluation of damage to the isolated cells in afferent elimination. ***A***, Low-magnification fluorescence images of dendrites and axons of the isolated target GFP-transfected cells before and 24 h after cutting. ***B***, Images in the upper row show axons outside of the cutting line (the green square in ***A***) with green arrowheads indicating disappearing axons. Images in the lower row show the soma and dendrites of the target neuron (the orange square in ***A***) with orange arrowheads indicating the preserved dendrite. Images before, 3, 6, and 24 h after cutting are presented. ***C***, Low-magnification images of an isolated target cell expressing GFP (green), together with phase contract image (left) or with anti-NF-H immunostaining (magenta, right). ***D***, Higher magnification images of the same neuron in (C) with GFP (green) and anti-NF-H (magenta) fluorescence signals inside of the region marked by a yellow square in ***C***. Yellow arrowheads indicate the axon, which starts from the target cell body and truncated at the intersection with the cutting line. Scale bars = 100 µm (***A***, ***C***), 10 µm (***B***), 50 µm (***D***). Download Extended Data 1, TIF file.

We next investigated whether reduction in local axonal components induced reduction of presynaptic boutons in contact with the target postsynaptic neurons. Single cell application of lipophilic dye DiI combined with immunostaining of presynaptic boutons by anti-bassoon antibody revealed the synaptic contact sites marked by presynaptic bassoon immunoreactivity and postsynaptic spine morphology. The rate of spines in contact with bassoon-positive presynaptic boutons was drastically reduced in the target cells deprived of axons compared to the rate in control cells at 24 h after afferent elimination [Fig. 1*F*; intact: 0.85 ± 0.04, *n* = 6 cells (3 h); 0.79 ± 0.04, *n* = 5 cells (6 h); 0.81 ± 0.05, *n* = 5 cells (24 h); cut: 0.82 ± 0.03, *n* = 3 cells (3 h); 0.60 ± 0.05, *n* = 7 cells (6 h); 0.11 ± 0.03, *n* = 4 cells (24 h); *p* > 0.999 (3 h), *p* = 0.011 (6 h), *p* < 0.001 (24 h), two-way ANOVA and Bonferroni’s multiple comparison test]. Evaluation of the fraction of spines without presynaptic bassoon puncta at 3 and 6 h after afferent elimination provided the information about the time course of presynapse elimination (Fig. 1*E*,*F*). Namely, loss of presynaptic structure was not evident at 3 h after axon cutting but there was a significant increase in the fraction of spines without associated bassoon-positive boutons at 6 h. These data indicate that afferent elimination can reduce the presynaptic sites making synaptic contacts with postsynaptic spines and this reduction in presynaptic structure starts at relatively early time points after manipulation.

### Afferent elimination induces loss of dendritic spines and postsynaptic scaffold protein

**Figure 2.**
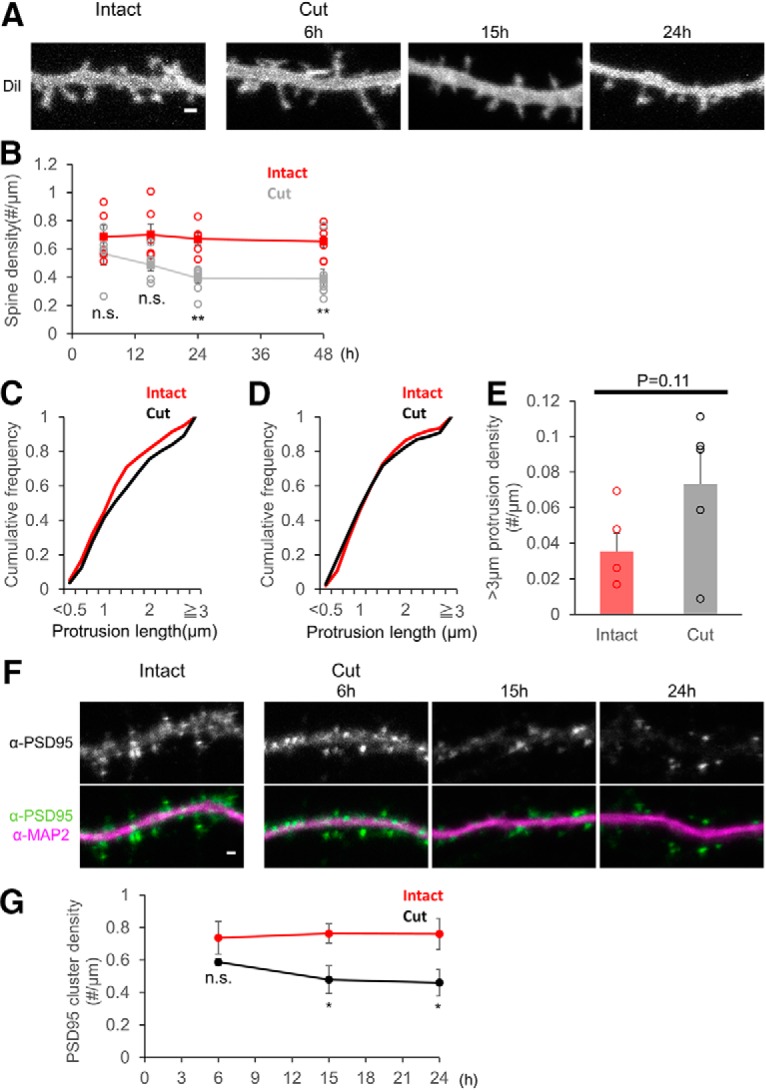
Time course of changes in spines after afferent axon cutting. ***A***, Confocal images of dendrites stained with DiI without afferent elimination (intact), or 6, 15, and 24 h after afferent elimination (cut). ***B***, Spine density at 6, 15, 24, and 48 h after afferent elimination. ***C***, ***D***, Cumulative frequency of spine lengths at 6 h (***C***) and 24 h (***D***) after afferent elimination. Spines after elimination of afferent axons showed a tendency to be longer than those in intact cells at 6 h. ***E***, Average densities of dendritic protrusions classified as long protrusion (>3 µm) in intact neurons or in neurons 6 h after afferent elimination. ***F***, Confocal images of dendrites immunostained with PSD-95 and MAP2 antibodies without afferent elimination (intact), or 6, 15, and 24 h after afferent elimination (cut). The upper row shows images of anti-PSD-95 immunofluorescence. The lower row shows merged immunofluorescence images of anti-PSD-95 (green) and anti-MAP2 (magenta). ***G***, Average densities of PSD-95 clusters along each dendrite at 6, 15, and 24 h after afferent elimination. Error bars are SEM; *p < 0.05, **p < 0.01, n.s. = not significant. Scale bars = 1 μm. Evaluation of damage by afferent axon elimination can be found in Extended Data Figure 2-1.

Majority of spines in the cortex and the hippocampus in adult mice are thought to contact with presynaptic sites (Yuste and Bonhoeffer, 2004). When presynaptic boutons are lost, concurrent spine loss may start. To investigate this possibility, we next measured spine density at multiple time points after afferent elimination. The spine density of the postsynaptic target neurons was maintained until 6 h after afferent elimination but started to decline at 15 h and further decreased to 60% of the control level at 24 and 48 h [Fig. 2*A*,*B*; intact: 0.69 ± 0.08/µm, *n* = 5 cells (6 h); 0.70 ± 0.08/µm, *n* = 6 cells (15 h); 0.67 ± 0.04/µm, *n* = 6 cells (24 h); 0.65 ± 0.04/µm, *n* = 7 cells (48 h); cut: 0.56 ± 0.08/µm, *n* = 5 cells (6 h); 0.49 ± 0.04/µm, *n* = 6 cells (15 h); 0.39 ± 0.03/µm, *n* = 7 cells (24 h); 0.39 ± 0.07/µm, *n* = 7 cells (48 h); *p* = 0.783 (6 h), *p* = 0.053 (15 h), *p* = 0.005 (24 h), *p* = 0.006 (48 h), two-way ANOVA and Bonferroni’s multiple comparison test]. Distribution of spine lengths showed a tendency of shift toward longer population transiently at 6 h after afferent elimination but returned to the distribution comparable to the control preparations at 24 h (Fig. 2*C–E*; intact: 0.035 ± 0.010, *n* = 5 cells; cut: 0.073 ± 0.018, *n* = 5 cells; *p* = 0.108, unpaired *t* test). The shift in spine or protrusion length distribution was mainly in the class of spines longer than 3 µm (Fig. 2*E*). Postsynaptic protein contents were expected to decline along with spine loss after afferent elimination. To confirm this, we measured cluster density of PSD-95, a major postsynaptic scaffolding protein, along dendrites by immunostaining at multiple time points after afferent elimination. Decline of PSD-95 cluster density was not clear at 6 h but was significant later than 15 h [Fig. 2*F*,*G*; intact: 0.74 ± 0.10/µm, *n* = 6 cells (6 h); 0.76 ± 0.06/µm, *n* = 7 cells (15 h); 0.76 ± 0.10/µm, *n* = 7 cells (24 h); cut: 0.58 ± 0.02/µm, *n* = 6 cells (6 h); 0.48 ± 0.08/µm, *n* = 7 cells (15 h); 0.46 ± 0.08/µm, *n* = 7 cells (24 h); *p* = 0.643 (6 h), *p* = 0.041 (15 h), *p* = 0.028 (24 h), two-way ANOVA and *post hoc* Bonferroni’s multiple comparison test]. The data revealed that elimination of spines and postsynaptic protein clusters started between 6 and 15 h after afferent elimination and lagged behind the presynaptic loss, which started 3 h after afferent elimination.

10.1523/ENEURO.0459-18.2019.f2-1 Extended Data Figure 2-1 Evaluation of damage induced by afferent elimination. ***A***, Phase-contrast and fluorescence images of a target cell with afferent elimination. The left panel shows a neuron before afferent elimination. The middle (merged image of phase contrast and PI fluorescence) and right (PI fluorescence image) panels show the neuron 6 h after cutting. PI staining detected the nuclei of the dead cells. Green arrowheads indicate injured cell bodies judged from phase-contrast images, while orange arrowheads indicate PI-positive cells. ***B***, Phase-contrast and immunofluorescence images of a target postsynaptic neuron with (cut) or without (intact) afferent elimination. The images of the target cell 6 h after afferent elimination were presented. MAP2 (dendritic marker) fluorescence intensity outside of the circular zone (yellow) and within 250 μm from the cutting line was measured. ***C***, Average fluorescence intensity of anti-MAP2 immunostaining was comparable between control (intact) and afferent elimination (cut). Error bars are SEM. Scale bars = 100 µm. Download Figure 2-1, TIF file.

If the damage to neighboring neurons is extensive, the observed effects of axon cutting to the postsynaptic target neurons may be explained by toxic substances released from injured cells. We therefore estimated the number of damaged cells outside of the cutting line. Estimation of dead cells in culture by incorporation of PI and phase-contrast image revealed that in control cell preparation without mechanical intervention, there were 7.5 ± 1.0/mm ^2^ (*n* = 5 areas) of dead neurons. In turn, we found 20.2 ± 6.2/mm^2^ (*n* = 5 areas) of dead cells in the area outside of the cutting line (within 250 μm from the cutting line). This increase of dead cell density is modest and limited to the area close to the cutting line, indicating limited amount of toxic substrates released from injured cells (Extended Data Fig. 2-1*A*). To evaluate the extent of the damage to the dendrites of neighboring cells, MAP2 immunostaining was performed at 24 h after cutting and MAP2 staining intensity was measured in a circular ring centered at the target neuron with its inner circumference defined by the cutting line and its outer circumference 250 µm from the center. As a control, we measured MAP2 fluorescence in a similar circular ring without the cutting procedure. We found no significant changes in MAP2 fluorescence intensity by the cutting procedure (Extended Data Fig. 2-1*B*,*C*; intact: 6.2 ± 1.0, *n* = 6 areas; cut: 6.4 ± 1.0, *n* = 6 areas; *p* = 0.887, unpaired *t* test). These data suggest that the damage to the neurons outside of the cutting line were limited to a small number of neurons and may not be sufficient to induce cell damage by released toxic substances.

### PSD clusters translocate toward dendritic shafts after afferent elimination

**Figure 3.**
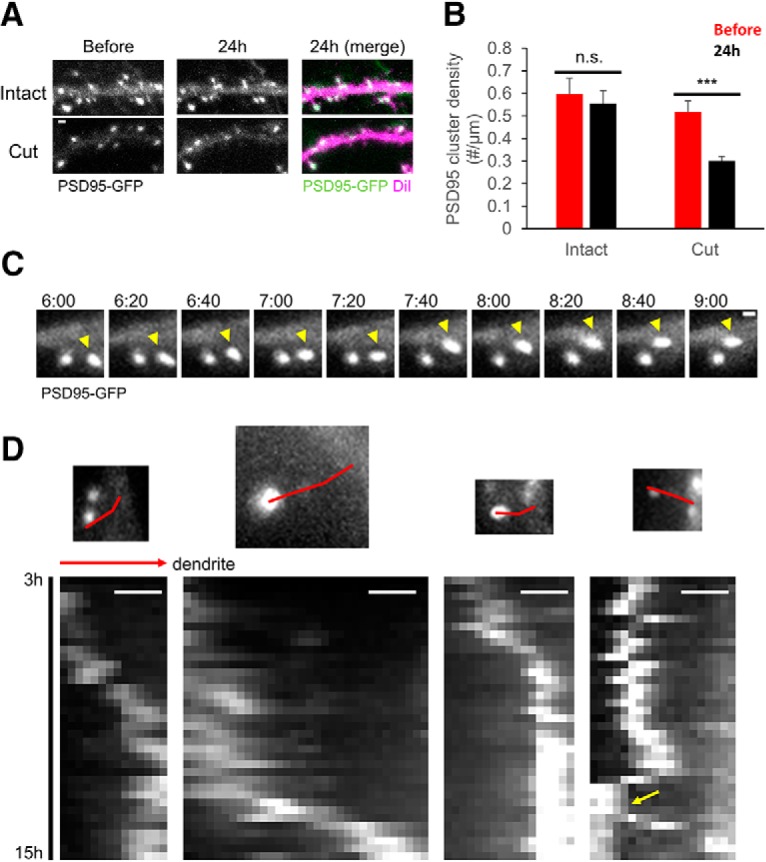
Temporal changes in PSD-95 clusters after afferent elimination. ***A***, Confocal images of dendrites with or without afferent elimination. Images of PSD-95-GFP in the same dendrites before and 24 h after the manipulation. The pseudo color images are overlay of PSD-95-GFP (green) and DiI (magenta) 24 h after the manipulation. ***B***, PSD-95-GFP cluster densities before and 24 h after afferent elimination. ***C***, Time-lapse images of a PSD-95-GFP cluster moving toward a dendritic shaft. Movement of the right cluster (yellow arrowheads) became evident at 7 h 40 min after the manipulation. ***D***, Kymographs of moving PSD-95-GFP clusters. Each image on top shows PSD-95-GFP clusters at 3 h after afferent elimination. A red line in each image on top shows the trajectory of cluster movement, along which the bottom kymograph was generated. Some PSD-95-GFP clusters split (a yellow arrow in the rightmost kymograph) and only one of the two clusters move toward the dendritic shaft. The kymographs start and end at 3 and 15 h after afferent elimination. Direction toward dendritic shafts are indicated by a red arrow. Error bars are SEM; ***p < 0.001, n.s. = not significant. Scale bars = 1 μm.

The delayed decrease in the density of postsynaptic spines after afferent elimination may induce gradual redistribution of postsynaptic molecules and translocation of PSDs. To characterize the temporal pattern in redistribution of PSD molecules and translocation of PSD clusters, we expressed PSD-95 tagged with GFP by infection of recombinant adenoviruses and repeated the manipulation of afferent elimination, followed by time-lapse imaging of fluorescent PSDs in living neurons. There was a significant reduction in the density of PSD-95-GFP clusters 24 h after afferent elimination [Fig. 3*A*,*B*; intact: 0.59 ± 0.07/µm, *n* = 5 cells (before); 0.55 ± 0.06/µm, *n* = 5 cells (24 h); cut: 0.51 ± 0.05/µm, *n* = 4 cells (before); 0.30 ± 0.02/µm, *n* = 4 cells (24 h); *p* = 0.275 (intact), *p* < 0.001 (cut), two-way ANOVA and *post hoc* Bonferroni’s multiple comparison test with repeated measures].

The reduction in PSD-95 clusters was associated with elimination of spines, which were identified by retrospective labeling of the postsynaptic neurons with DiI (Fig. 3*A*). To further characterize the relationship between PSD cluster remodeling and spine elimination, we performed live cell imaging of PSD-95-GFP with shorter time intervals of 20 min spanning the time window from 3 to 15 h, in which significant decline of spine density should take place (Fig. 3*C*,*D*). We could identify multiple events of PSD movement toward dendritic shafts at the average speed of 3.20 ± 0.71 µm/h (*n* = 10 clusters). The initial time frames of PSD movement were at 6–7 h after afferent elimination. There were also events of PSD split into two clusters while moving (Fig. 3*D*, image set at the right end with a yellow arrow).

**Figure 4.**
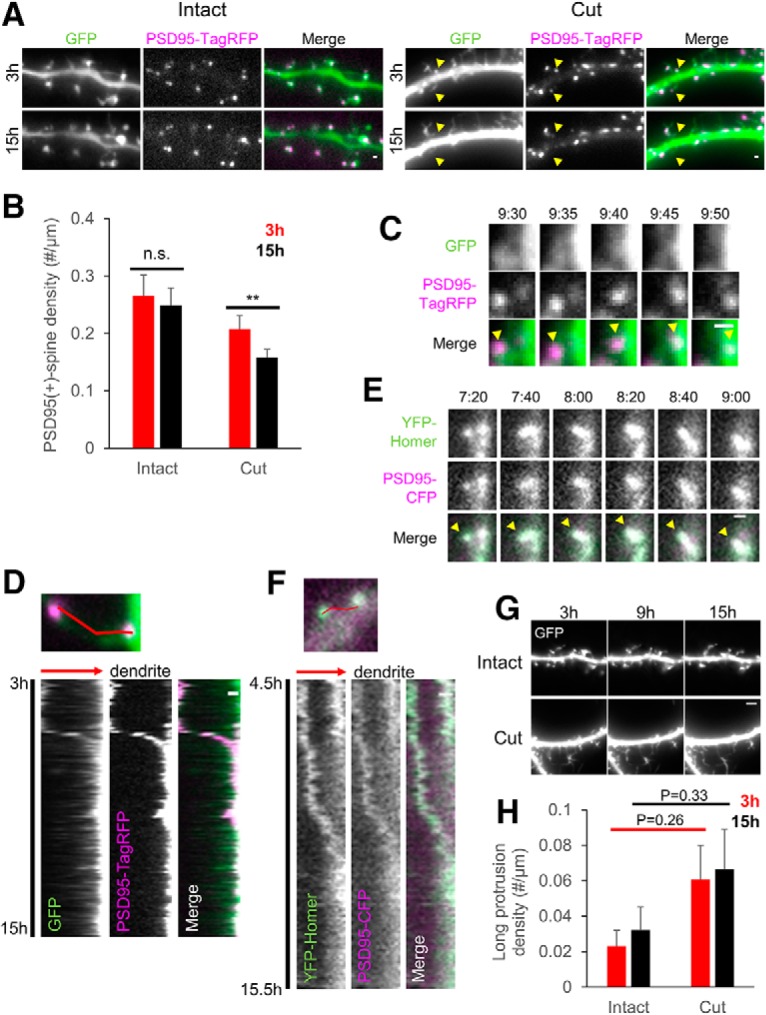
Temporal changes in PSD-95-positive spines after afferent elimination. ***A***, Time-lapse images of dendrites with or without afferent elimination 3 and 15 h after manipulation. GFP, PSD-95-TagRFP, and their merged images (GFP; green, PSD-95-TagRFP; magenta) from left to right, respectively. Yellow arrowheads indicate loss of PSD-95-positive spines. ***B***, Densities of PSD-95-positive spines 3 and 15 h after afferent elimination. ***C***, Representative time-lapse sequences of a PSD-95-TagRFP cluster moving toward a dendritic shaft. The upper row shows time-lapse images of GFP. The middle row shows time-lapse images of PSD-95-TagRFP. The lower row in ***C*** is merged images of GFP (green) and PSD-95-TagRFP (magenta) with yellow arrowheads indicating translocating clusters. ***D***, The top image shows a PSD-95-TagRFP cluster in the spine before moving. Along a red line in the image, the kymograph was generated. Each column in the kymograph shows the images of GFP, PSD-95-TagRFP or merged images of GFP (green) and PSD-95-TagRFP (magenta) from left to right, respectively. The kymographs start and end at 3 and 15 h after afferent elimination. The direction of the dendritic shaft is indicated by a red arrow. ***E***, Representative image sequences of YFP-Homer1c (top) and PSD-95-CFP (middle) clusters moving together toward a dendritic shaft, together with an overlay of YFP-Homer1c (green) and PSD-95-CFP (magenta). Yellow arrowheads indicate a translocating cluster. ***F***, The top image shows YFP-Homer1c and PSD-95-CFP clusters before moving. Along a red line in the image, kymographs were created. Each column in the kymographs shows YFP-Homer1c, PSD-95-CFP or overlay of YFP-Homer1c (green) and PSD-95-CFP (magenta) from left to right, respectively. The kymographs start and end at 4.5 and 15.5 h after afferent elimination. Direction toward the dendritic shaft is indicated by a red arrow. ***G***, Time-lapse imaging of GFP-expressing dendrites with or without afferent elimination at 0 h and subsequent data acquisition at 3, 9, and 15 h. ***H***, Density of dendritic protrusions classified as long protrusions (>4 µm in lengths) at 3 and 15 h after afferent elimination. Error bars are SEM; **p < 0.01, n.s. = not significant. Scale bars = 1 μm (***A***, ***C–F***), 4 μm (***G***). Methods for axon-preserving alternate afferent axon elimination can be found in Extended Data Figure 4-1, and Results from alternate afferent axon elimination can be found in Extended Data Figure 4-2.

Remodeling of PSDs after afferent elimination may be associated with spine remodeling and initiation of new protrusions from dendritic shafts. To identify relationship between PSD remodeling and dendritic protrusive activity, dual color live imaging of postsynaptic neurons should be performed. For this purpose, we expressed both GFP and PSD-95-TagRFP by chemical transfection and repeated the procedure of afferent elimination. With this protocol combined with dual color live imaging of both GFP and PSD-95, we found decline in the density of PSD-95-positive spines from 3 to 15 h after afferent elimination [Fig. 4*A*,*B*; intact: 0.27 ± 0.04/µm, *n* = 6 cells (3 h); 0.25 ± 0.03/µm, *n* = 6 cells (15 h); cut: 0.21 ± 0.02/µm, *n* = 6 cells (3 h); 0.15 ± 0.01/µm, *n* = 6 cells (15 h); *p* = 0.402 (intact), *p* = 0.004 (cut), two-way ANOVA and *post hoc* Bonferroni’s multiple comparison test with repeated measures]. In three-quarters of the events of PSD-95 loss in time-lapse imaging, PSD-95 clusters showed translocation toward the dendritic shafts (Fig. 4*C*,*D*; 72.5 ± 5.0%, *n* = 4 cells). In most cases, these events of PSD-95 translocation took place simultaneously with spine shrinkage, detected by GFP imaging. Simultaneous imaging of PSD-95-CFP and YFP-Homer1c, another prominent postsynaptic scaffolding molecule, revealed simultaneous translocation of both molecules (Fig. 4*E*,*F*), indicating the PSD-95 translocation events reported structural changes in the PSD molecular assembly.

10.1523/ENEURO.0459-18.2019.f4-1 Extended Data Figure 4-1 Afferent axon elimination with the axon from the target neuron preserved. ***A***, ***B***, An experimental setup of afferent axon cutting in dissociated hippocampal neuron culture. A micromanipulator-assisted cutting system on an inverted fluorescence microscopy with phase-contrast illumination (***A***), which enables fine movement of a needle (***B***, tip diameter of 0.30 mm) for precise control of the cutting trajectory on the culture surface. ***C***, Overlay of phase-contrast and fluorescence images of a target cell (red arrow) before and after afferent elimination (yellow arrowheads). ***D***, Lower magnification fluorescence images which include the area shown in ***C***. Anti-bassoon immunoreactivity of the target cell (red arrows) was lower than that of neurons outside of the cutting line. Scale bars = 200 μm (***C***, ***D***). Download Figure 4-1, TIF file.

10.1523/ENEURO.0459-18.2019.f4-2 Extended Data Figure 4-2 Changes in PSD-95 clusters after afferent elimination without transection of the axon growing from the target neuron. ***A***, Confocal images of GFP (green) and PSD-95-TagRFP (magenta) with or without afferent elimination. ***B***, Relative decrease in the density of PSD-95-positive spines 24 h after afferent elimination. ***C***, Confocal images of GFP (green) and anti-bassoon clusters (magenta) with or without afferent elimination. ***D***, Fractions of bassoon-positive spines in the total spine population 24 h after afferent elimination. ***E***, A representative time-lapse sequence of two PSD-95-TagRFP clusters moving toward a dendritic shaft. The upper time-lapse sequence shows dynamics of PSD-95-TagRFP clusters. The lower time-lapse sequence is merged images of GFP (green) and PSD-95-TagRFP (magenta). White arrows indicate the first translocating cluster from spines to the dendritic shaft. Red arrows indicate the second PSD-95 cluster split into two clusters in the process of translocation. Error bars are SEM; **p < 0.01, ***p < 0.001. Scale bars = 2 μm (***A***, ***C***), 1 μm (***E***). Download Figure 4-2, TIF file.

Damage of afferent axons and subsequent reduction of PSD structure and spines may induce up-regulation in protrusive activity of filopodia-like structures from dendrites (Fig. 4*G*). Quantitative analysis revealed that this protrusive activity was two to three times higher at both 3 and 15 h after afferent elimination in comparison with the control condition, but without statistical significance [Fig. 4*G*,*H*; intact: 0.023 ± 0.008/µm, *n* = 6 cells (3 h); 0.032 ± 0.013/µm, *n* = 6 cells (15 h); cut: 0.061 ± 0.019/µm, *n* = 6 cells (3 h); 0.066 ± 0.022/µm, *n* = 6 cells (15 h); *p* = 0.262 (3 h), *p* = 0.331 (15 h), two-way ANOVA and *post hoc* Bonferroni’s test]. Possible upregulation of dendritic protrusive activity at 3 h after afferent elimination suggests that this early dendritic change is not triggered by elimination of presynaptic activity, which was not evident at this time point.

Our method of mechanical transection of incoming axons inevitably transect the efferent axon from the target postsynaptic neuron (Extended Data Fig. 1-1*C*,*D*). To evaluate the effect of axon transection to the postsynaptic neuron, we searched the culture dishes to identify the efferent axon extending from the target postsynaptic neuron and damaged this axon by applying the minimal mechanical deformation. This procedure induced subsequent degeneration of the efferent axon without detectable changes in the density of surrounding incoming axons. Quantification of spine densities in the postsynaptic neurons confirmed no obvious change between 3 and 15 h (3h: 0.20 ± 0.02/µm, *n* = 5 cells; 15 h: 0.19 ± 0.03/µm, *n* = 5 cells; *p* = 0.858, unpaired *t* test). These data suggest that the translocation of PSD-95 clusters or the loss of PSD-95-positive spines did not occur by efferent axon elimination of the target postsynaptic neurons.

To further clarify that afferent elimination is sufficient to induce changes in postsynaptic structures of the target neuron, we took another approach to preserve the axons extending from the target neuron while eliminating most of the afferent axons (Extended Data Fig. 4-1). Consistent with the results after transecting both the afferent and efferent axons, the density of PSD-95-positive spines was significantly reduced at 24 h after selective afferent transection (Extended Data Fig. 4-2*A*,*B*; intact: 1.01 ± 0.05, *n* = 3 cells; cut: 0.62 ± 0.09, *n* = 3 cells; *p* = 0.002, unpaired *t* test). We also confirmed reduction of presynaptic structures with this protocol (Extended Data Fig. 4-2*C*,*D*; intact: 0.92 ± 0.03/µm, *n* = 3 cells; cut: 0.11 ± 0.01, *n* = 3 cells; *p* < 0.001, unpaired *t* test). Next, we performed live cell imaging of GFP and PSD-95-TagRFP from 3 to 15 h after the selective afferent transection. Translocation of PSD-95 clusters to dendritic shafts was again confirmed in this experimental condition (Extended Data Fig. 4-2*E*). These results support the idea that selective afferent elimination is sufficient to induce loss of PSD-95 from spines in the postsynaptic target neurons.

### Loss of presynaptic sites precedes translocation of PSD clusters after afferent elimination

**Figure 5.**
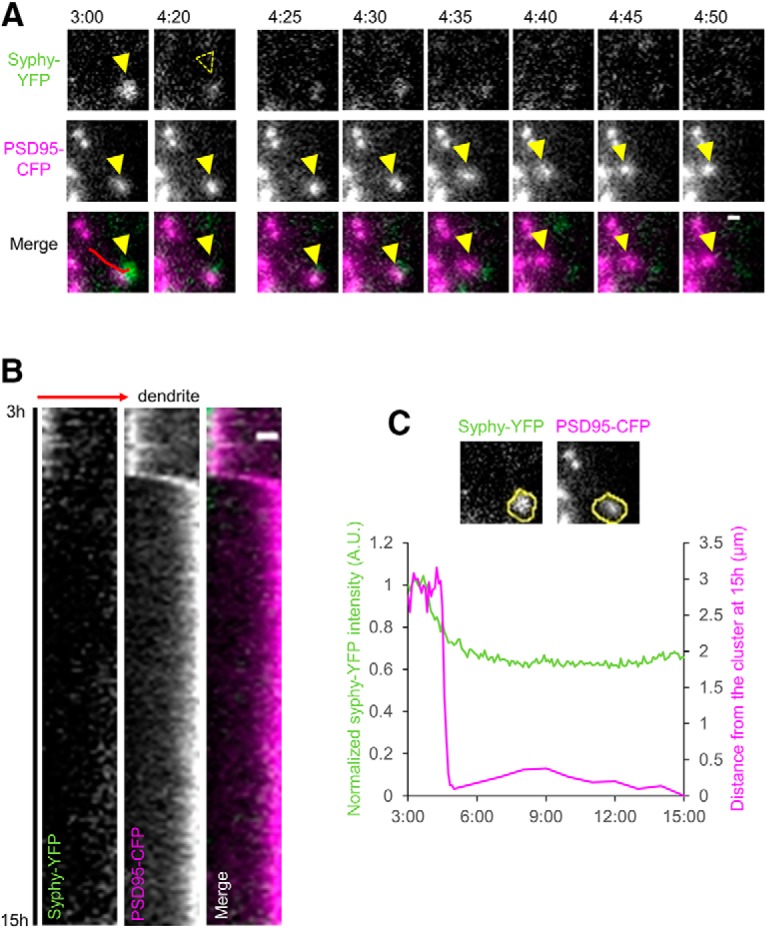
Simultaneous imaging of presynaptic and postsynaptic sites. ***A***, Time-lapse imaging of a PSD-95-CFP cluster moving toward a dendritic shaft after disappearance of a synaptophysin-YFP cluster in contact with the PSD-95-CFP cluster. Merged images (synaptophysin-YFP; green, PSD-95-CFP; magenta) are shown in the bottom row. Yellow arrowheads indicate adjacent synaptophysin-YFP and PSD-95-CFP clusters and elimination of the synaptophysin-YFP cluster at 4 h 20 min and subsequent translocation of the PSD-95-CFP cluster starting at 4 h 30 min after afferent elimination. ***B***, Kymographs created along the red line in the merged image at 3 h in ***A***. Each column of the kymographs shows synaptophysin-YFP, PSD-95-CFP, and their merged images (synaptophysin-YFP; green, PSD-95-CFP; magenta) from left to right, respectively. The kymographs start and end at 3 and 15 h after afferent elimination. The direction toward the dendritic shaft is indicated by a red arrow. ***C***, Images of a synaptophysin-YFP cluster and a PSD-95-CFP cluster at 3 h after afferent elimination with yellow ROIs set to quantitate fluorescent intensities of the synaptophysin-YFP cluster (green line in the graph) and translocation distance of the PSD-95-CFP cluster measured from the final position at 15 h after cutting (magenta line in the graph). Error bars are SEM. Scale bars = 1 μm.

The temporal profiles of decrease in presynaptic structures (Fig. 1*E*) and postsynaptic spines (Fig. 2*B*) indicate that loss of presynaptic boutons precedes loss of postsynaptic spines. To confirm the temporal order of synapse deconstruction, we expressed synaptophysin-YFP, the marker of synaptic vesicles, and PSD-95-CFP by recombinant adenoviruses. Time-lapse imaging was performed from 3 to 15 h after afferent elimination with time intervals of 5 min (Fig. 5*A*). Several examples of synapse loss were identified from the time-lapse sequences, including an example of presynapse elimination that took place from 3 to 7 h after afferent elimination, followed by gradual translocation of the postsynaptic PSD-95 cluster and disappearance of the spine structure (Fig. 5*A–C*). Temporal gaps between the loss of synaptophysin-YFP clusters and the start of PSD-95-CFP cluster movement were variable (from 10 min to 9 h). There were few cases in which the movement of PSD-95-CFP clusters initiated with intact synaptophysin-YFP clusters. The data suggest that spine shrinkage and associated translocation of PSDs are triggered by preceding loss of presynaptic structure.

### Spine loss induced by afferent elimination is rescued by inhibition of NMDA receptor activity

**Figure 6.**
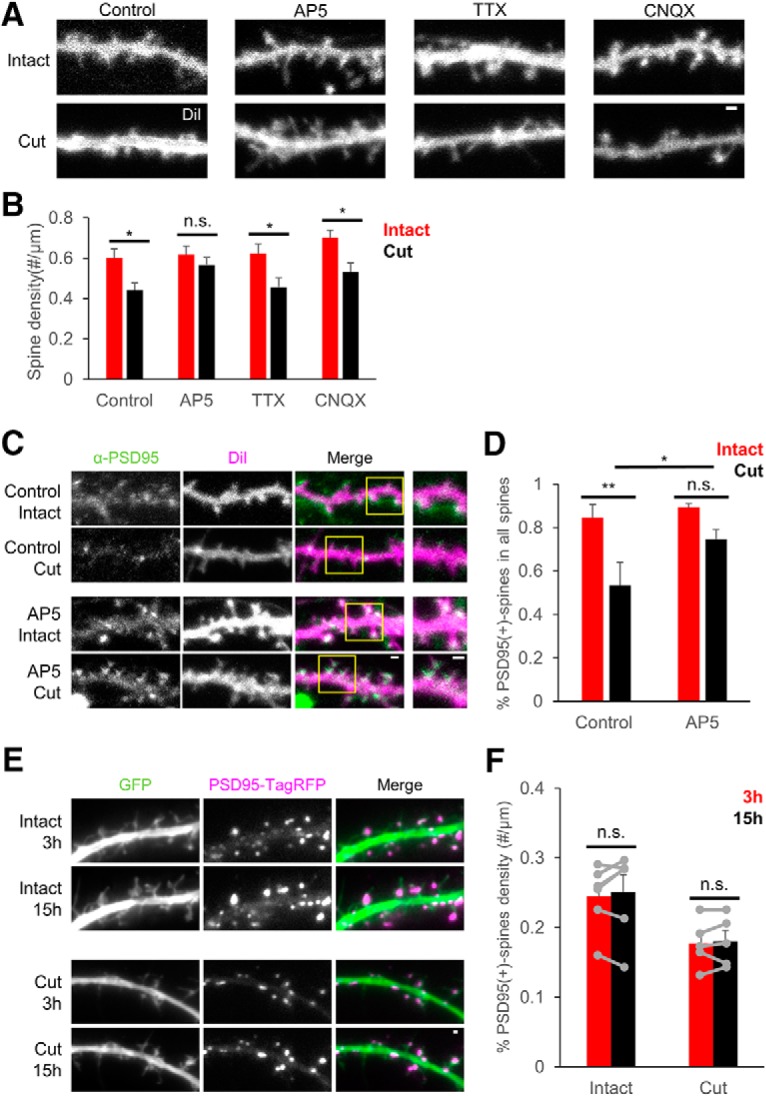
Pharmacological manipulation of neuronal activity and postsynaptic function after afferent elimination. ***A***, Confocal images of dendrites stained with DiI 24 h after afferent elimination with or without administration of either AP5, TTX, or CNQX. ***B***, The effects of AP5, TTX, and CNQX on spine density 24 h after afferent elimination. ***C***, Confocal images of dendrites with or without AP5 administration 24 h after afferent elimination. As a control, neurons without afferent elimination were also treated with AP5. Images in each row show anti-PSD-95 immunostaining, DiI labeling, and their merged images (PSD-95; green, DiI; magenta) from left to right. The rightmost column shows enlarged images of the areas marked by yellow squares. ***D***, Fractions of PSD-95-positive spines in the total spine population in conditions with or without AP5 and with or without afferent elimination. Spines were imaged 24 h after afferent elimination. ***E***, Time-lapse images of dendrites with (cut) or without (intact) afferent elimination at 3 and 15 h under AP5 administration. Afferent elimination was performed at 0 h. GFP, PSD-95-TagRFP, and their merged images (GFP; green, PSD-95-TagRFP; magenta) from left to right, respectively. ***F***, Densities of PSD-95-positive spines at 3 and 15 h with or without afferent elimination in the presence of AP5. Error bars are SEM; *p < 0.05, **p < 0.01, n.s. = not significant. Scale bars = 1 μm. Effect of AP5 to the presynaptic sites can be found in Extended Data Figure 6-1.

In our reduced model of afferent elimination in culture, the postsynaptic response to the manipulation may mimic the pathway that operates after TBI. Previous reports indicate that the overstimulation of NMDA receptors plays an important role in acceleration of pathologic changes induced by TBI (Morrison et al., 2011; Shohami and Biegon, 2014). Therefore, we next evaluated the effect of applying blockers for NMDA receptors, AMPA receptors, and voltage-gated sodium channels for reduction of postsynaptic changes after afferent elimination. Although AMPA receptor blocker CNQX and voltage-gated sodium channel blocker TTX did not prevent spine elimination, NMDA receptor blocker AP5 was effective in suppressing the deteriorative effects of afferent elimination [Fig. 6*A*,*B*; intact: 0.60 ± 0.05/µm, *n* = 9 cells (control); 0.61 ± 0.04/µm, *n* = 9 cells (AP5); 0.62 ± 0.05/µm, *n* = 9 cells (TTX); 0.70 ± 0.04/µm, *n* = 10 cells (CNQX); cut: 0.44 ± 0.03/µm, *n* = 10 cells (control); 0.56 ± 0.03/µm, *n* = 12 cells (AP5); 0.46 ± 0.05/µm, *n* = 10 cells (TTX); 0.53 ± 0.04/µm, *n* = 10 cells (CNQX); *p* = 0.038 (control), *p* > 0.999 (AP5), *p* = 0.020 (TTX), *p* = 0.029 (CNQX), two-way ANOVA and *post hoc* Bonferroni’s multiple comparison test]. Quantification of the density of PSD-95-positive spines after afferent elimination with or without NMDA receptor blocker AP5 confirmed the protective effect of AP5 on PSD-95-positive spines, while the effect was partial in terms of the comparison with the condition without afferent elimination [Fig. 6*C*,*D*; control (without AP5): 0.84 ± 0.04/µm, *n* = 5 cells (intact); 0.53 ± 0.08/µm, *n* = 7 cells (cut); AP5: 0.89 ± 0.02/µm, *n* = 6 cells (intact); 0.74 ± 0.04/µm, *n* = 7 cells (cut); *p* = 0.003 (intact vs cut without AP5), *p* = 0.210 (intact vs cut with AP5), *p* = 0.030 (without vs with AP5), two-way ANOVA and *post hoc* Tukey’s test]. Quantification of the density of PSD-95-positive spines in live-imaging also revealed protective effect of AP5 in the reduction of PSD-95-positive spines during the period of 3–15 h after afferent elimination [Fig. 6*E*,*F*; intact: 0.24 ± 0.02/µm, *n* = 6 cells (3 h); 0.25 ± 0.02/µm, *n* = 5 cells (15 h); cut: 0.18 ± 0.02/µm, *n* = 5 cells (3 h); 0.18 ± 0.02/µm, *n* = 5 cells (15 h); *p* = 0.789 (intact), *p* > 0.999 (cut), two-way ANOVA and *post hoc* Bonferroni’s multiple comparison test with repeated measures]. On the other hand, AP5 was not effective in preventing the reduction of bassoon immunopositive puncta induced by afferent elimination [Fig. 1*F*; Extended Data Fig. 6-1; 3 h: 0.82 ± 0.08/µm, *n* = 5 cells (intact); 0.89 ± 0.24/µm, *n* = 5 cells (cut); 6h: 1.09 ± 0.18/µm, *n* = 5 cells (intact); 0.48 ± 0.09/µm, *n* = 5 cells (cut); *p* > 0.999 (3 h), *p* = 0.037 (6 h), two-way ANOVA with *post hoc* Bonferroni’s multiple comparison test]. Together, these data suggest that blockade of NMDA receptors selectively prevented loss of spines and PSD-95 clusters.

10.1523/ENEURO.0459-18.2019.f6-1 Extended Data Figure 6-1 Temporal changes in bassoon clusters after afferent elimination combined with AP5 administration. ***A***, Confocal images of dendrites with bassoon and MAP2 immunofluorescence either without axon manipulation (intact), or 3 and 6 h after afferent elimination (cut). The top and third rows show anti-bassoon immunoreactivity. The second and fourth rows show double-staining with anti-bassoon (green) and anti-MAP2 (magenta). ***B***, Average densities of bassoon clusters along dendrites 3 and 6 h after afferent elimination. Error bars are SEM; *p < 0.05. Scale bars = 1 μm. Download Figure 6-1, TIF file.

## Discussion

This study aimed to reveal synaptic changes following afferent elimination, a procedure that mimics axonal damage resulting from TBI. First, we showed that the procedure was able to reduce the fraction of spines in contact with presynaptic boutons (Fig. 1). Spines and PSD-95 clusters, the postsynaptic marker, were also reduced following afferent elimination, but the reduction in the presynaptic sites started earlier than the reduction of the spines (Fig. 2). PSD-95 clusters were also reduced and some clusters moved toward dendritic shafts (Figs. 2–4). This translocation may be associated with spine retraction, since moving PSD-95 clusters were observed in about half of the lost spines (Fig. 4). Also, the loss of presynaptic sites showed a temporal relationship with PSD-95 cluster translocation in simultaneous imaging of presynaptic and postsynaptic molecules (Fig. 5). AP5, an antagonist of NMDA receptors, was able to partially rescue this decrease in both total spines and PSD-95-positive spines, suggesting that the postsynaptic events, including spine retraction and PSD-95 cluster translocation, are NMDA receptor-dependent (Fig. 6).

In TBI, axons are subjected to mechanical damage and these damaged axons subsequently undergo the process of Wallerian degeneration (Armstrong et al., 2016). Also, the numbers of synaptophysin clusters and synaptophysin expression level has been reported to decrease in animal models of TBI (Thompson et al., 2006; Tchantchou et al., 2017). Our afferent elimination procedure performed in culture was able to reduce the number of presynaptic sites by mechanical axonal injury and induce axonal degeneration (Fig. 1), suggesting that our culture system can reflect certain aspects of axon degeneration that occur following TBI. A device designed to induce axonal injury without damage to the neuron cell bodies (Taylor et al., 2005) was previously used in experiments designed to characterize morphology of axotomized cells (Nagendran et al., 2017). However, axotomy could only be applied to axons located in microgrooves between the two chambers of the device; this device configuration limited axotomy to the axons coming from the opposite chamber only. The precise time course of the decrease in presynaptic bouton density *in vivo* following TBI remains unknown. Axon fragmentation or proteolysis of neurofilaments begins ∼ 6 h after axotomy in cultured dorsal root ganglion neurons (Gerdts et al., 2016). This timing is in line with our observation of a decline in presynaptic boutons following the manipulation. This temporal gap between axonal injury and loss of presynaptic structures may provide a possible time window of opportunity for manipulation aimed at preventing the axonal damage.

Spines and postsynaptic sites also decrease in animal models of TBI (Wakade et al., 2010; Campbell et al., 2012; Winston et al., 2013; Tchantchou et al., 2017), but simultaneous damage to both presynaptic axons and postsynaptic neurons could not be distinguished in animal models of TBI (Gao et al., 2011; Gao and Chen, 2011). Also, the contribution of glial cells, which are in close contact with neuronal components, especially synaptic structures, should be considered in TBI models (Eroglu and Barres, 2010). Indeed, previous studies have reported the involvement of glia-derived secreted factors that can affect synaptic density or synaptic plasticity following TBI or after stroke (Liauw et al., 2008; Perez et al., 2017). Here, we used afferent elimination in a primary hippocampal culture and this procedure was able to keep postsynaptic neurons intact and free from the effects of glial cells, because our dissociated cell culture system contained few glial cells. However, we scratched the dish and damaged the culture surface, which prevented the growth of new axons across the scratched surface, resulting in the entry of few new axons to the zone of axon elimination. This situation may be problematic, as the entry of new axons starts within a few days of injury in animal models (Nudo, 2013). Our afferent elimination procedure should be improved to replicate the phase of axon reentry and initiation of new circuit formation.

In our experimental conditions, the axotomy of target cells did not induce spine loss, while afferent elimination without transection of axons originating from the target neuron was sufficient to induce spine loss (Extended Data Fig. 4-2). These results strongly suggest that spine loss was induced by presynaptic elimination, and not by axotomy of the postsynaptic neuron. A previous study using dissociated hippocampal neuron culture reported a 20% decrease in spine density in cells 24 h after axotomy (Nagendran et al., 2017). In this previous study, neurons were cultured in two compartments, axons extending from one compartment were transected, and the effect on the postsynaptic neurons was assessed in the same compartment. It is likely that afferent transection affected most of the postsynaptic neurons in this experimental setup and may have induced population-level changes in the neuron culture. In contrast, in our procedure we manipulated a single neuron while preserving most of the other neurons, leaving them intact. These differences may explain the minimal effects of axotomy on postsynaptic neurons in our experimental protocols. Netrin-1 signaling has been postulated to be involved in synaptic remodeling following massive axotomy (Nagendran et al., 2017) and this signaling cascade may not be activated by our transection procedure.

There were two main phenomena observed in isolated cells after afferent elimination: an increase in long dendritic protrusions and the translocation of PSD clusters toward dendrites. The time course of presynaptic loss induced by afferent elimination (Fig. 1) overlapped with that of the increase in long dendritic protrusions (Figs. 2*C–E*, 4*G*,*H*). These results suggest that long protrusions, such as filopodia and spine head protrusions, emerge in response to a loss of presynaptic activity. Dendritic filopodia are thought to play a role as explorers, searching for new axonal partners by virtue of their length and mobility (Yuste and Bonhoeffer, 2004; Ozcan, 2017). Therefore, we hypothesize that the long dendritic protrusions may search for new axonal partners to compensate for any presynaptic losses. Together with presynaptic losses, we observed the translocation of PSD clusters toward dendrites, following afferent elimination (Figs. 3–5). When PSD clusters translocated, most spines containing these clusters also shrank in relation to the translocation (Fig. 4*C*; Extended Data Fig. 4-2*E*). This means that spine loss induced by afferent elimination is closely linked with postsynaptic loss in most cases. Together, we speculate that the phenomena observed in isolated cells following afferent elimination reflect synaptic plasticity in cells after TBI. Further studies may discover the key to promoting synaptic recovery by controlling this plasticity.

Our experiments suggest that both spine loss and PSD translocation are NMDA receptor dependent and can be blocked by NMDA receptor antagonists (Fig. 6). Previous reports also showed that blocking NMDA receptors could prevent neuronal death (Rao et al., 2001) or improve learning and memory abilities (Han et al., 2009) in rat models of TBI. However, clinical trials of NMDA antagonists for treating TBI in humans were unsuccessful (Shohami and Biegon, 2014). This may be explained by the heterogeneity in human populations or inadequate outcome measures (Shohami and Biegon, 2014), but may also be due to complex responses of the injured nervous system to therapeutic agents. For example, the activation of NMDA receptors may have neurotoxic effects during the acute phase of TBI, but may be neuroprotective in later phases (Ikonomidou and Turski, 2002). Indeed, the administration of D-cycloserine, an NMDA receptor partial co-agonist, was shown to improve functional outcomes over a wide temporal window in a mouse model of TBI (Adeleye et al., 2010). In our experiments, NMDA receptor antagonists could not completely block the loss of PSD-95-positive spines (Fig. 6*C*,*D*), suggesting that other factors might also influence the survival of spine synapses. The reduced culture system we developed in this study may be useful for dissecting the complex responses of the neural network following injury and determining the appropriate timing of therapeutic interventions.

## Synthesis

Reviewing Editor: Mike Fainzilber, Weizmann Institute of Science Decisions are customarily a result of the Reviewing Editor and the peer reviewers coming together and discussing their recommendations until a consensus is reached. When revisions are invited, a fact-based synthesis statement explaining their decision and outlining what is needed to prepare a revision will be listed below. The following reviewer(s) agreed to reveal their identity: Anna Karpova.

In their revised manuscript entitled, “Temporal sequences of synapse corruption triggered by afferent axon transection - time-lapse imaging study of pre- and post-synaptic molecules” Authors have clarified the consequences of in vitro mechanical injury of hippocampal primary neurons that resulted in loss of presynaptic sites that preceded the loss of postsynaptic sites and showed that this effect can be attenuated by blockade of NMDA receptors.

We appreciate the authors efforts for improving the manuscript. They adequately and satisfactorily responded to all comments, questions and concerns and introduced the necessary changes to the manuscript.

Although the revised manuscript has been improved, some polishing could still improve it further. For example the authors might consider implications of the finding that PSD-95 as well as Homer1c relocates into dendritic shaft as a cluster upon axonal elimination and loss of spine structure induced up-regulation in protrusive activity of filipodia-like structures from dendrites. Why is this not discussed? If the authors think that this is a compensatory mechanism, how this can be utilized by the system?

Suggestions regarding Figures:

- Image labeling inside a box often masks some information. Examples: Figure 1B, Figure 2F; Figure 3A; Figure 3C; figure 4A (3h and 15h), 4C and 4E; Figure 1-1C and D; Figure 2-1 A and B; Figure 4-1 C and D; Figure 4-2 A and C; Figure 6-1.

-Please keep some space between image frames : Figure 5A; Figure 4C,E, D, F.

Figure 1A; 4-2E; - scale bars are missing.

Results section:

-Subtitles in the Results section should bring the message about the finding and not about an experimental approach.

- The arguments in the discussion could be improved, perhaps with the help of an editing service.

## Author Response

Responses to the reviewers:

1. Although the revised manuscript has been improved, some polishing could still improve it further. For example the authors might consider implications of the finding that PSD-95 as well as Homer1c relocates into dendritic shaft as a cluster upon axonal elimination and loss of spine structure induced up-regulation in protrusive activity of filipodia-like structures from dendrites. Why is this not discussed? If the authors think that this is a compensatory mechanism, how this can be utilized by the system?

Thank you so much for the synthesis comments. Following the comments, we added discussion about relationship between spine loss and up-regulation in protrusive activity as below (lines 503-519):

“There were two main phenomena observed in isolated cells after afferent elimination: an increase in long dendritic protrusions and the translocation of PSD clusters toward dendrites. The time course of presynaptic loss induced by afferent elimination (Fig. 1) overlapped with that of the increase in long dendritic protrusions (Fig. 2C-E, 4G and 4H). These results suggest that long protrusions, such as filopodia and spine head protrusions, emerge in response to a loss of presynaptic activity. Dendritic filopodia are thought to play a role as explorers, searching for new axonal partners by virtue of their length and mobility (Yuste and Bonhoeffer, 2004; Ozcan, 2017). Therefore, we hypothesize that the long dendritic protrusions may search for new axonal partners to compensate for any presynaptic losses. Together with presynaptic losses, we observed the translocation of PSD clusters towards dendrites, following afferent elimination (Fig. 3-5). When PSD clusters translocated, most spines containing these clusters also shrank in relation to the translocation (Fig. 4C and Extended data Fig. 4-2 E). This means that spine loss induced by afferent elimination is closely linked with postsynaptic loss in most cases. Together, we speculate that the phenomena observed in isolated cells following afferent elimination reflect synaptic plasticity in cells after TBI. Further studies may discover the key to promoting synaptic recovery by controlling this plasticity.”

2-1. Image labeling inside a box often masks some information. Examples: Figure 1B, Figure 2F; Figure 3A; Figure 3C; figure 4A (3h and 15h), 4C and 4E; Figure 1-1C and D; Figure 2-1 A and B; Figure 4-1 C and D; Figure 4-2 A and C; Figure 6-1.

Texture labeling was moved outside the box in all listed figures. Labeling by figures like arrows or arrowheads was also moved or changed to avoid masking information in some figures.

2-2. Please keep some space between image frames: Figure 5A; Figure 4C, E, D, F.

We made some space between images in Figure 4C, E, D, F and 5A.

2-3. Figure 1A; 4-2E; - scale bars are missing.

We added scale bars to Figure 1A. For Figure 4-2E, we omitted scale bars in upper rows because upper rows and lower rows show the same place.

3. Subtitles in the Results section should bring the message about the finding and not about an experimental approach.

We changed subtitles in the Results sections as the subtitles bring the message about the findings (lines 223, 266, 313-314, 387, 403).

4. The arguments in the discussion could be improved, perhaps with the help of an editing service.

Following your advice, we used an editing service and improved the discussion.

5. Additional modification.

In addition to these modifications, we changed the title of our manuscript from “Temporal sequences of synapse corruption triggered by afferent axon transection - time-lapse imaging study of pre- and post-synaptic molecules” to “Temporal sequences of synapse disintegration triggered by afferent axon transection - time-lapse imaging study of pre- and post-synaptic molecules”, as the word “corruption” is not adequate to describe gradual loss of molecular and structural elements from synapses.
