# Supplementary figures and images for: Temporal Sequences of Synapse Disintegration Triggered by Afferent Axon Transection, Time-Lapse Imaging Study of Presynaptic and Postsynaptic Molecules
Source: eNeuro. 2019 Oct 2;6(5):ENEURO.0459-18.2019. doi: 10.1523/ENEURO.0459-18.2019 (PMC6785539; doi:10.1523/ENEURO.0459-18.2019)

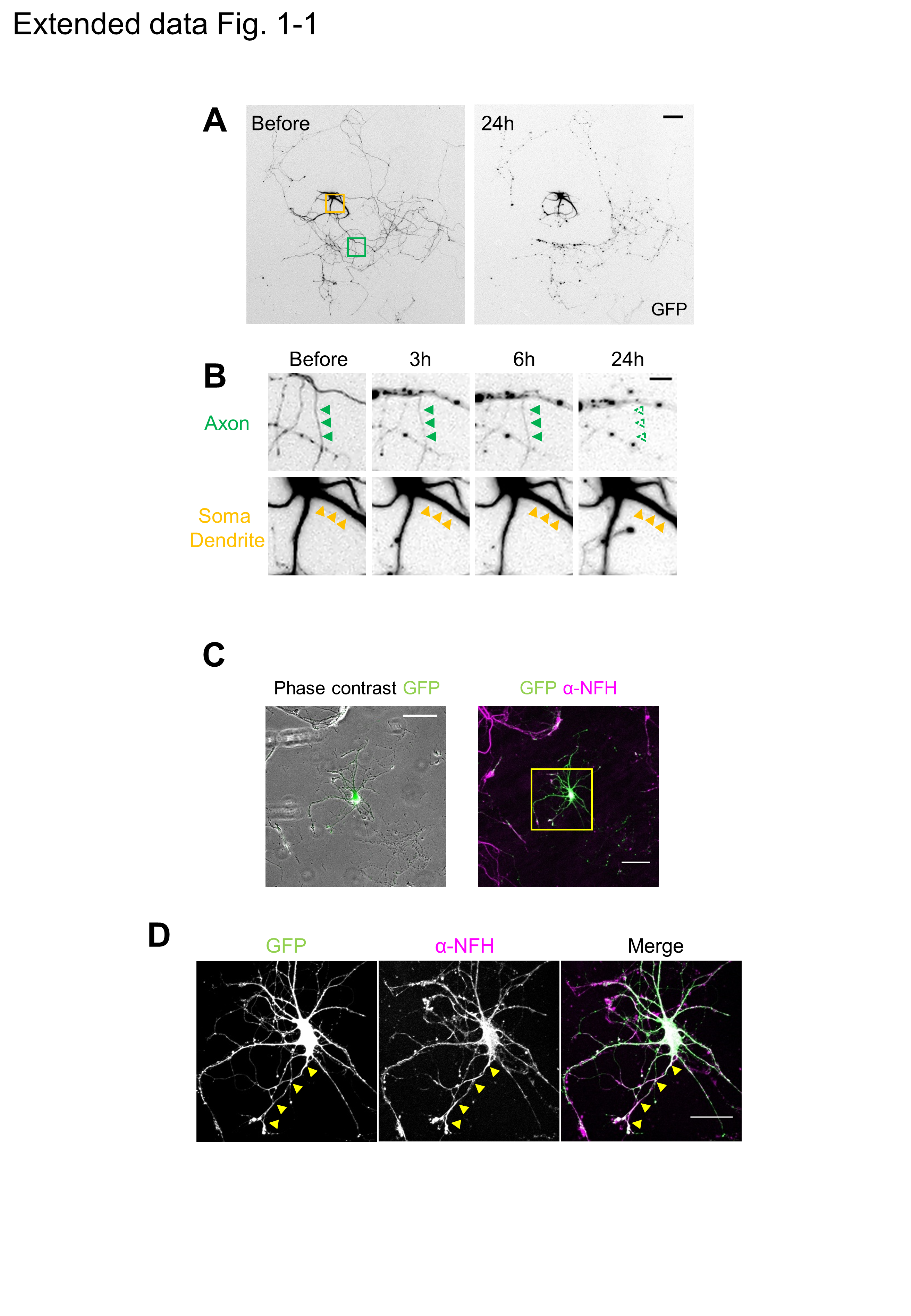

Supplement: Extended Data Figure 1-1. — Evaluation of damage to the isolated cells in afferent elimination. A, Low-magnification fluorescence images of dendrites and axons of the isolated target GFP-transfected cells before and 24 h after cutting. B, Images in the upper row show axons outside of the cutting line (the green square in A) with green arrowheads indicating disappearing axons. Images in the lower row show the soma and dendrites of the target neuron (the orange square in A) with orange arrowheads indicating the preserved dendrite. Images before, 3, 6, and 24 h after cutting are presented. C, Low-magnification images of an isolated target cell expressing GFP (green), together with phase contract image (left) or with anti-NF-H immunostaining (magenta, right). D, Higher magnification images of the same neuron in (C) with GFP (green) and anti-NF-H (magenta) fluorescence signals inside of the region marked by a yellow square in C. Yellow arrowheads indicate the axon, which starts from the target cell body and truncated at the intersection with the cutting line. Scale bars = 100 µm (A, C), 10 µm (B), 50 µm (D). Download Extended Data 1, TIF file. [file sup_enu-eN-NWR-0459-18-s01.tif]

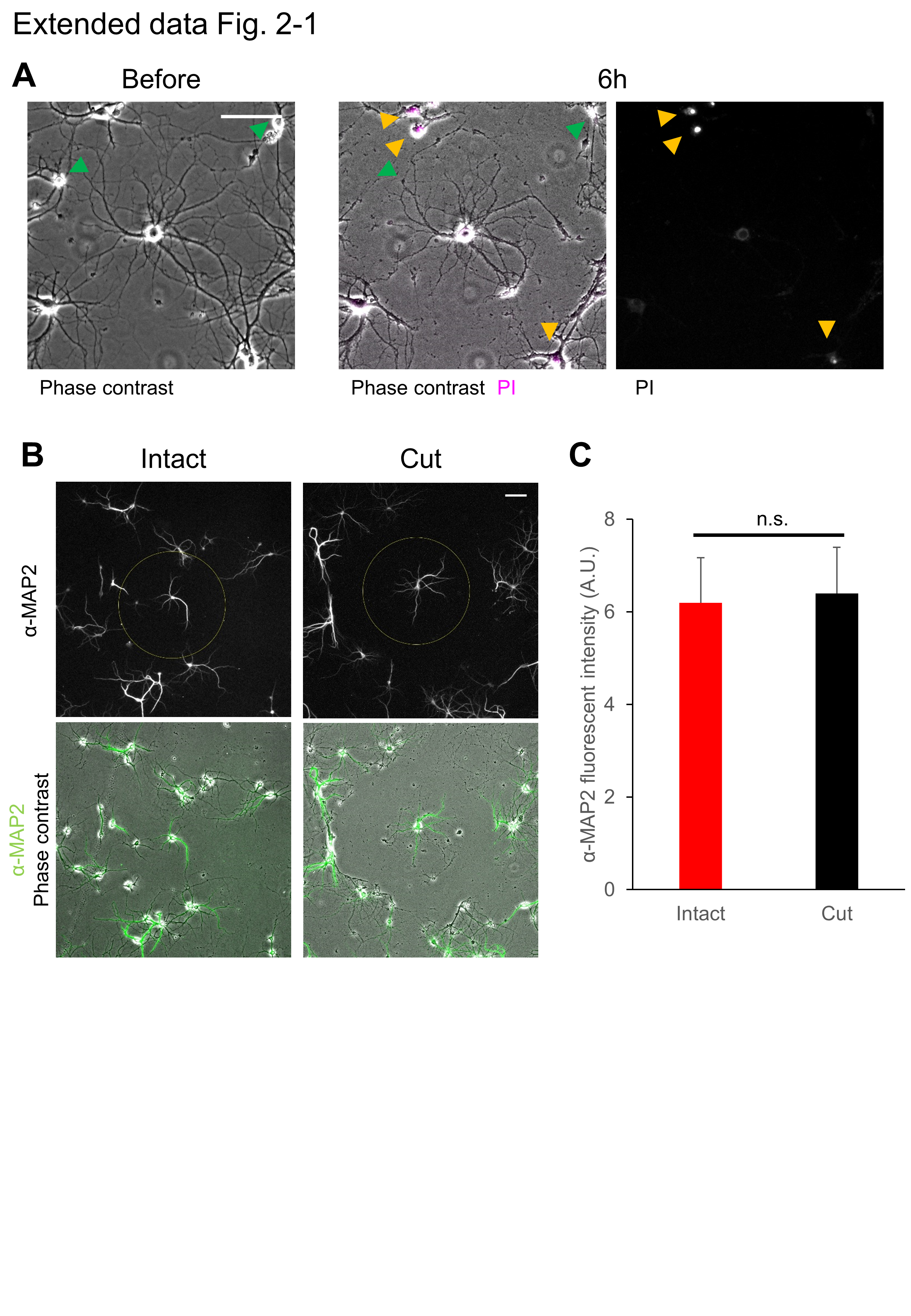

Supplement: Extended Data Figure 2-1 — Evaluation of damage induced by afferent elimination. A, Phase-contrast and fluorescence images of a target cell with afferent elimination. The left panel shows a neuron before afferent elimination. The middle (merged image of phase contrast and PI fluorescence) and right (PI fluorescence image) panels show the neuron 6 h after cutting. PI staining detected the nuclei of the dead cells. Green arrowheads indicate injured cell bodies judged from phase-contrast images, while orange arrowheads indicate PI-positive cells. B, Phase-contrast and immunofluorescence images of a target postsynaptic neuron with (cut) or without (intact) afferent elimination. The images of the target cell 6 h after afferent elimination were presented. MAP2 (dendritic marker) fluorescence intensity outside of the circular zone (yellow) and within 250 μm from the cutting line was measured. C, Average fluorescence intensity of anti-MAP2 immunostaining was comparable between control (intact) and afferent elimination (cut). Error bars are SEM. Scale bars = 100 µm. Download Figure 2-1, TIF file. [file sup_enu-eN-NWR-0459-18-s02.tif]

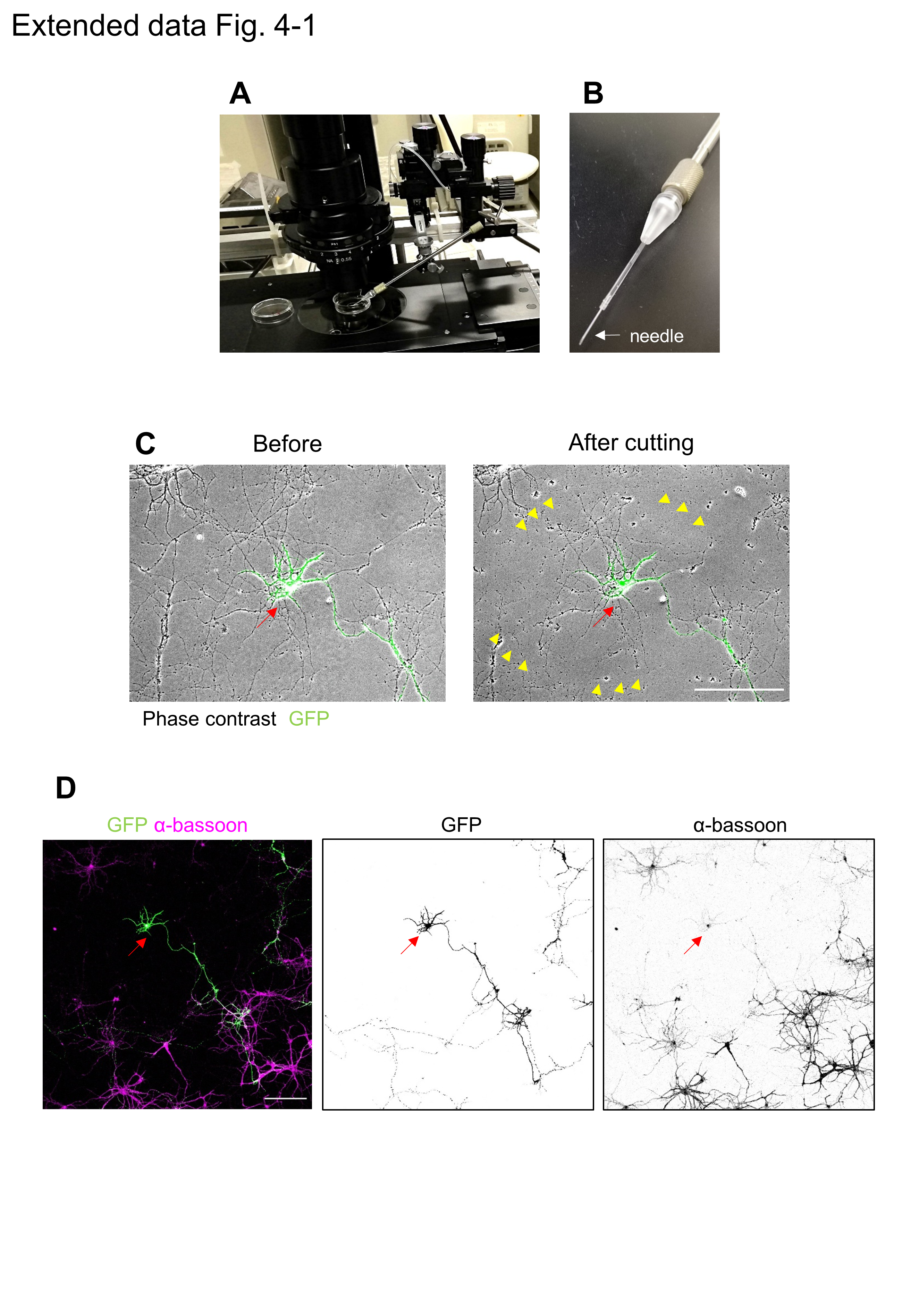

Supplement: Extended Data Figure 4-1 — Afferent axon elimination with the axon from the target neuron preserved. A, B, An experimental setup of afferent axon cutting in dissociated hippocampal neuron culture. A micromanipulator-assisted cutting system on an inverted fluorescence microscopy with phase-contrast illumination (A), which enables fine movement of a needle (B, tip diameter of 0.30 mm) for precise control of the cutting trajectory on the culture surface. C, Overlay of phase-contrast and fluorescence images of a target cell (red arrow) before and after afferent elimination (yellow arrowheads). D, Lower magnification fluorescence images which include the area shown in C. Anti-bassoon immunoreactivity of the target cell (red arrows) was lower than that of neurons outside of the cutting line. Scale bars = 200 μm (C, D). Download Figure 4-1, TIF file. [file sup_enu-eN-NWR-0459-18-s03.tif]

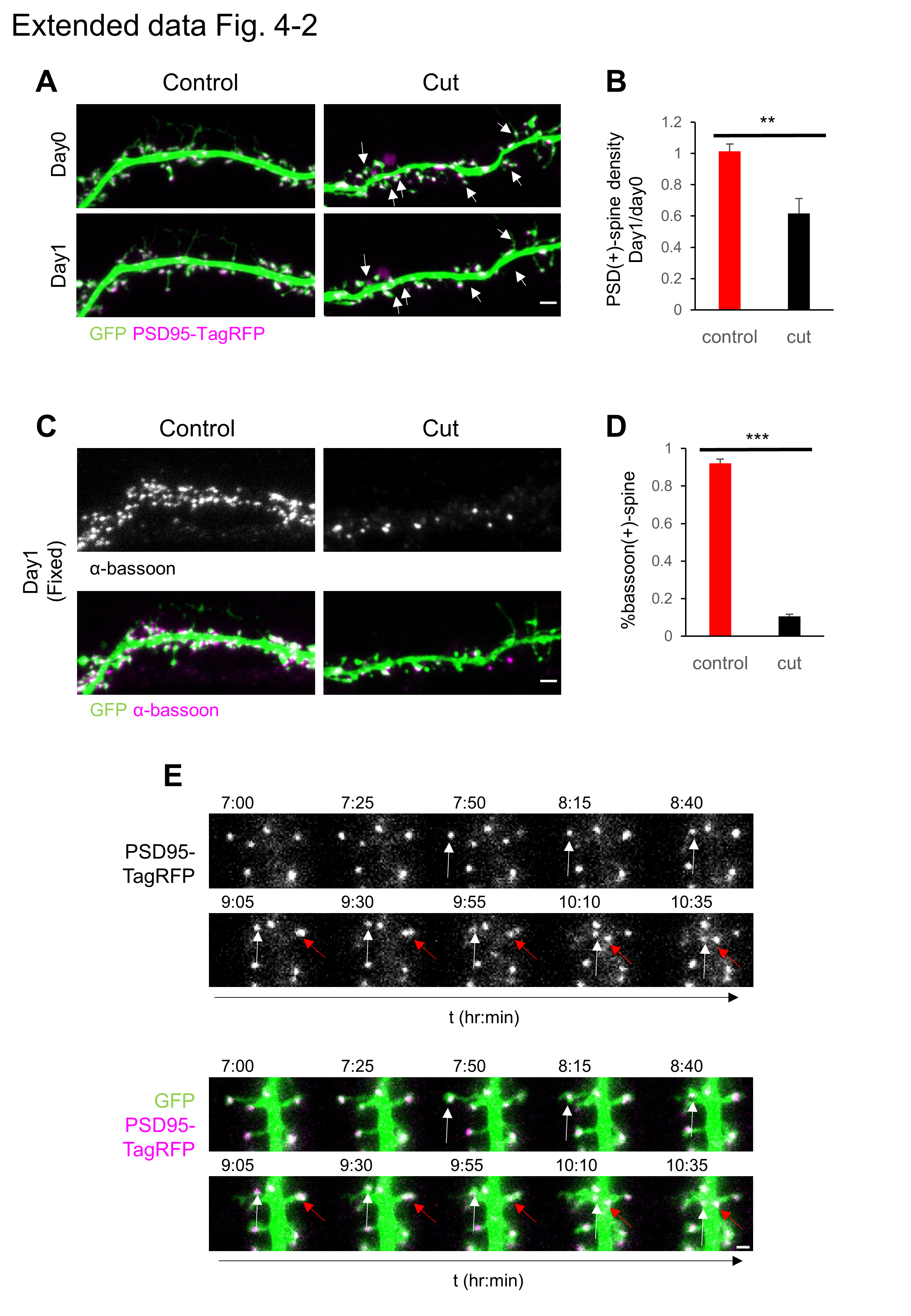

Supplement: Extended Data Figure 4-2 — Changes in PSD-95 clusters after afferent elimination without transection of the axon growing from the target neuron. A, Confocal images of GFP (green) and PSD-95-TagRFP (magenta) with or without afferent elimination. B, Relative decrease in the density of PSD-95-positive spines 24 h after afferent elimination. C, Confocal images of GFP (green) and anti-bassoon clusters (magenta) with or without afferent elimination. D, Fractions of bassoon-positive spines in the total spine population 24 h after afferent elimination. E, A representative time-lapse sequence of two PSD-95-TagRFP clusters moving toward a dendritic shaft. The upper time-lapse sequence shows dynamics of PSD-95-TagRFP clusters. The lower time-lapse sequence is merged images of GFP (green) and PSD-95-TagRFP (magenta). White arrows indicate the first translocating cluster from spines to the dendritic shaft. Red arrows indicate the second PSD-95 cluster split into two clusters in the process of translocation. Error bars are SEM; **p < 0.01, ***p < 0.001. Scale bars = 2 μm (A, C), 1 μm (E). Download Figure 4-2, TIF file. [file sup_enu-eN-NWR-0459-18-s04.tif]

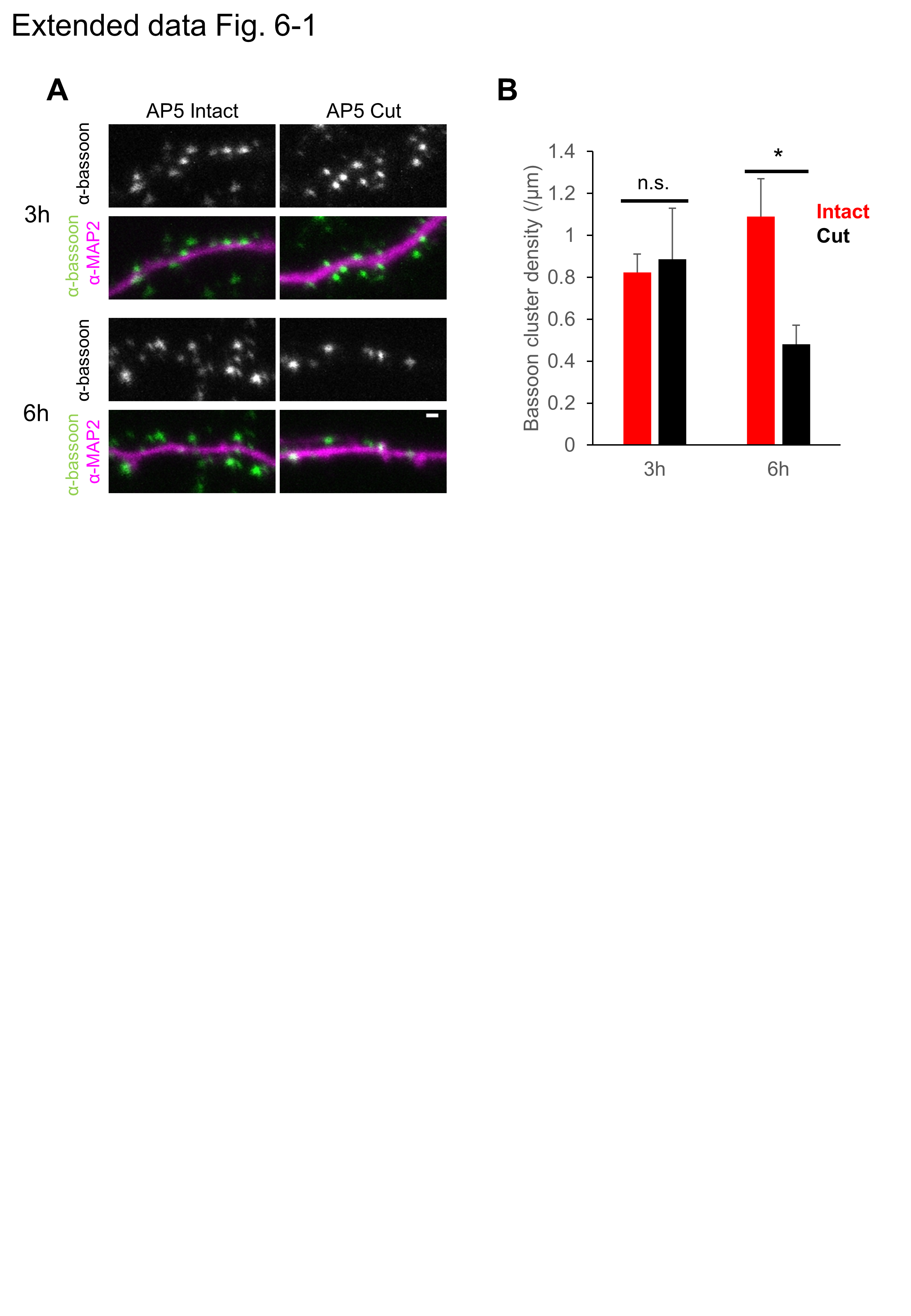

Supplement: Extended Data Figure 6-1 — Temporal changes in bassoon clusters after afferent elimination combined with AP5 administration. A, Confocal images of dendrites with bassoon and MAP2 immunofluorescence either without axon manipulation (intact), or 3 and 6 h after afferent elimination (cut). The top and third rows show anti-bassoon immunoreactivity. The second and fourth rows show double-staining with anti-bassoon (green) and anti-MAP2 (magenta). B, Average densities of bassoon clusters along dendrites 3 and 6 h after afferent elimination. Error bars are SEM; *p < 0.05. Scale bars = 1 μm. Download Figure 6-1, TIF file. [file sup_enu-eN-NWR-0459-18-s05.tif]
